# *Bacillus subtilis* RarA forms damage-inducible foci that scan the entire cell

**DOI:** 10.1186/s13104-019-4252-x

**Published:** 2019-04-11

**Authors:** Rogelio Hernández-Tamayo, Peter L. Graumann

**Affiliations:** 1 SYNMIKRO, LOEWE Center for Synthetic Microbiology, Marburg, Germany; 2 Department of Chemistry, Philipps Universität Marburg, Hans-Meerwein-Straße 6, 35032 Marburg, Germany

**Keywords:** RarA, DNA repair, Time lapse fluorescence microscopy, *Bacillus subtilis*, Stress response

## Abstract

**Objectives:**

Little is known about the activity and dynamics of ATPase RarA in *B. subtilis*, proposed to act at stalled DNA replication forks due to DNA damage. We performed fluorescence microscopy time lapse experiments with a functional RarA-mVenus fusion to visualize the dynamics of RarA during conditions that generate DNA damage.

**Data description:**

In exponentially growing cells, we observed that 15% of the cells contained single RarA-mV (mVenus fluorescent fusion) foci moving throughout the entire cell between 3 min intervals. This percentage remained constant at different time points, indicating that focus formation during unperturbed growth is maintained at about a constant rate. When cells were exposed to stress conditions, the population of cells containing RarA-mV foci tripled after 60 min. Cells exposed to two DNA-damaging drugs, to 5 mM MMS or to 0.5 mM H_2_O_2_, showed a similar type of response, with RarA-mVenus foci moving more slowly than during unperturbed growth. It is likely that RarA-mV contributes to the repair of H_2_O_2_-induced lesions, and to a minor extent to MMS-induced lesions. The presence of foci in growing cells suggests that RarA also plays a role during the cell cycle, at least in a fraction of cells, possibly contributing to heterogeneity of response to DNA damage.

## Objective

The bacterial replication-associated recombination protein A, RarA, belongs to a highly conserved family of ATPases, including the yeast Mgs1 and mammal WRNIP1 proteins [1]. The *B. subtilis rarA* gene, which is monocistronic, is constitutively expressed, but its expression is markedly enhanced by stressors such as diamide, ethanol, high salt or H_2_O_2_ [2]. RarA protein plays an important, but poorly understood role in genome maintenance [3]. Although several studies agreed with the idea that RarA acts in both replication and recombination processes, the concrete function is still unknown. *E. coli* RarA, which is co-expressed with FtsK, co-localizes/interacts with SeqA, RecQ [4], UvrD [5] or RecA [6] and may act at blocked forks in certain replication mutants [5, 6]. In vitro, *E. coli* RarA interacts with single strand binding (SSB) protein and shows helicase activity that preferentially unwinds 3′-ends from dsDNA ends or ssDNA gaps, suggesting that RarA could act at stalled replication forks [1, 7]. One common point of RarA studies is the complex scenario required to produce a clear phenotype that explains all observations.

## Data description

A C-terminal fusion of the fluorescent protein mVenus to RarA was generated by cloning the 3′-end 500 bp of *rarA* (excluding the stop codon) into plasmid pSG1164 [8], which was integrated into the *rarA* gene locus on the *B. subtilis* chromosome by homologous recombination. We have used epifluorescence microscopy time-lapse to monitor foci formation and dynamics of RarA before and after stress conditions at 30 °C (OD_600_ = ~ 0.3). Cells were either treated with 0.5 mM H_2_O_2_, or with 5 mM MMS (both obtained from Sigma Aldrich) or were not treated. For fluorescence microscopy, *B. subtilis* cells were grown in S7_50_ minimal medium [9] at 30 °C under shaking conditions until exponential growth. Three microliters of cells were transferred on an agarose slide—a glass slide (microscope slides standard, Roth) coated with an agarose layer (S7_50_ minimal medium, 1% v/v agarose) and covered with a cover slip (Roth). Fluorescence microscopy was performed using a Zeiss Observer Z1 (Carl Zeiss) with an oil immersion objective (100× magnification, NA 1.45 alpha Plan-FLUAR) and a CCD camera (CoolSNAP EZ, Photometrics), or with a BX51 microscope (Olympus) with a Cool Snap EZ camera (Photometrics) and a xenon light source (Olympus). Electronic data were processed using Metamorph 7.5.5.0 software (Molecular Devices, Sunnyvale, CA, USA), which also allows the calibration of the fluorescence intensity and pixel size to determine the cell length, time-lapse epifluorescence microscopy of RarA-mV were collected every 3 min.

In epifluorescence, an accumulation of fluorescent molecules is needed for detection, so it is reasonable to say that in exponentially growing cells, and to a higher extent in response to drugs that produce DNA damage, RarA is recruited to mobile assemblies within the cell. In case of induced DNA damage, RarA is assembled into foci in twice to three times as many cells than under exponential growth conditions (Table 1). The intensity of the response, considered as the increase of the percentage of cells containing RarA-mV foci, was 100% higher after MMS (from 15 to 30%, n = 125), and H_2_O_2_ addition produced an increase in the population of cells containing foci to about 40% of all cells imaged (n = 120). Movies 1 to 3 [10–12] show that RarA-mVenus foci moved throughout the cells with no apparent spatial specificity (Table 1, data file 1–3). As under exponential growth conditions [13], RarA-mVenus foci in hydrogen peroxide-stressed cells moved continuously with stochastic halts, and moved through the entire space of the cell. In about 10% of the cells containing foci, these appeared at some time point of the experiment or disappeared; in the remaining cells, foci were continuously present. Visually, movement of RarA could not be distinguished between stressed and non-stressed cells, merely the number of cells containing foci increased in cells repairing induced damage. However, automated tracking of focus movement and Gaussian mixture model (GMM) analyses (Data set 1) [14] showed two Gaussian distributions, corresponding to a slower/static and a faster/mobile fraction of RarA-mV assemblies, with diffusion constants of *D*_*static*_ = 3.12 µm^2^ min^−1^ or *D*_*mobile*_ = 31.8 µm^2^ min^−1^, under different growth conditions. Analyses of dynamics of single particles and determination of static and mobile fractions were performed using the Matlab-based graphical user interphase program SMTracker [15]. Compared to unperturbed growth, movement of RarA-mV became considerably slower after addition of MMS or H_2_O_2_: in contrast to 78% dynamic and 22% slow/static foci during exponential growth, MMS-treated cells showed 34% dynamic and 66% static foci, and H_2_O_2_-treated cells 36% dynamic and 64% static foci. RarA molecules never arrested for many minutes but continued scanning the cell, and were much longer-lived than e.g. RecN foci [16].

**Table 1:** Overview of data files/data sets

Label	Name of data file/data set	File types (file extension)	Data repository and identifier (DOI or accession number)
Data file 1 [10]	RarA-mV WT	Time lapse AVI	10.6084/m9.figshare.7461587.v3
Data file 2 [11]	RarA-mV MMS	Time lapse AVI	10.6084/m9.figshare.7461692.v2
Data file 3 [12]	RarA-mV H_2_O_2_	Time lapse AVI	10.6084/m9.figshare.7461698.v2
Data set 1 [14]	Gaussian mixture model (GMM) RarA-mV	Image tif	10.6084/m9.figshare.7466987.v3

## Limitations

This study extends observation of RarA-mVenus foci during unperturbed growth [13]. The study reveals the movement of an assembly of RarA molecules in a subset of a cell population; it does not describe the dynamics of freely diffusing molecules. Although clearly, foci are only present in a minority of cells, even after stress induction, very small assemblies may be present in more cells, but may be undetectable through epifluorescence microscopy.

## Abbreviations

Mgs1: maintenance of genome stability 1
WRNIP1: Werner [WRN] Interacting Protein 1
MMS: methyl methane sulfonate
H_2_O_2_: peroxide water
GMM: Gaussian mixture model
